# Supplementary material for: Structure of a cleavage-independent HIV Env recapitulates the glycoprotein architecture of the native cleaved trimer
Source: Nat Commun. 2018 May 16;9:1956. doi: 10.1038/s41467-018-04272-y (PMC5955915; doi:10.1038/s41467-018-04272-y)
Supplement: Supplementary file 1 — Supplementary Information [file 41467_2018_4272_MOESM1_ESM.pdf]

Supplementary Information for

Structure of a cleavage-independent HIV Env recapitulates the glycoprotein architecture of the native cleaved trimer

Sarkar et al.

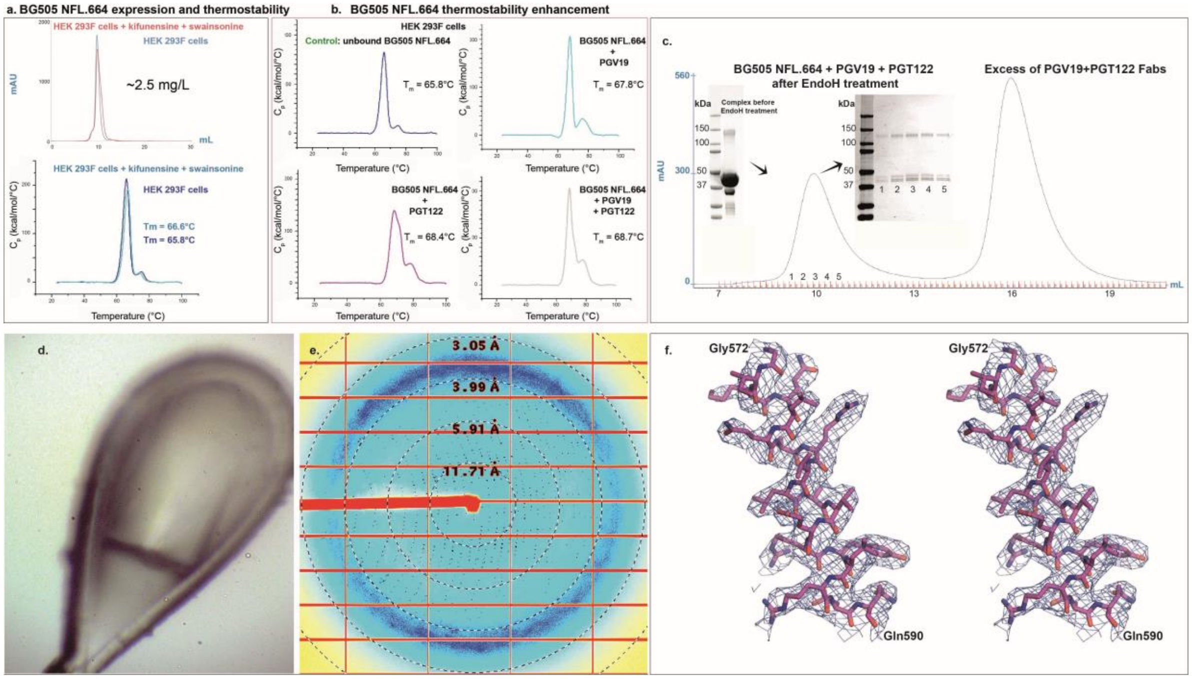

Supplementary Figure 1. BG505 NFL.664 expression, thermostability and crystallization. (a) The top panel compares the expression profiles of BG505 NFL.664 when expressed in HEK 293F cells with (pink) or without (blue) glycosidase inhibitors (kifunensine and swainsonine). The lower panel compares the thermostability of the proteins in the top panel. **(b)** Thermostability of BG505 NFL.664 expressed in HEK 293F cells and its stabilization from binding of Fabs PGV19 and PGT122. **(c)** A complex of BG505 NFL.664 Env was formed with PGV19 and PGT122 in a 1:2 ratio per binding site, followed by EndoH digestion and SEC purification. **(d)** The crystal obtained from the purified complex described in (c). **(e)** A frame representing X-ray diffraction from the crystal in (d). **(e)** A stereo representation of the *2Fo-Fc* electron density map at 1.0σ for a section of the HR1$_C$ region of the BG505 NFL.664 complex.

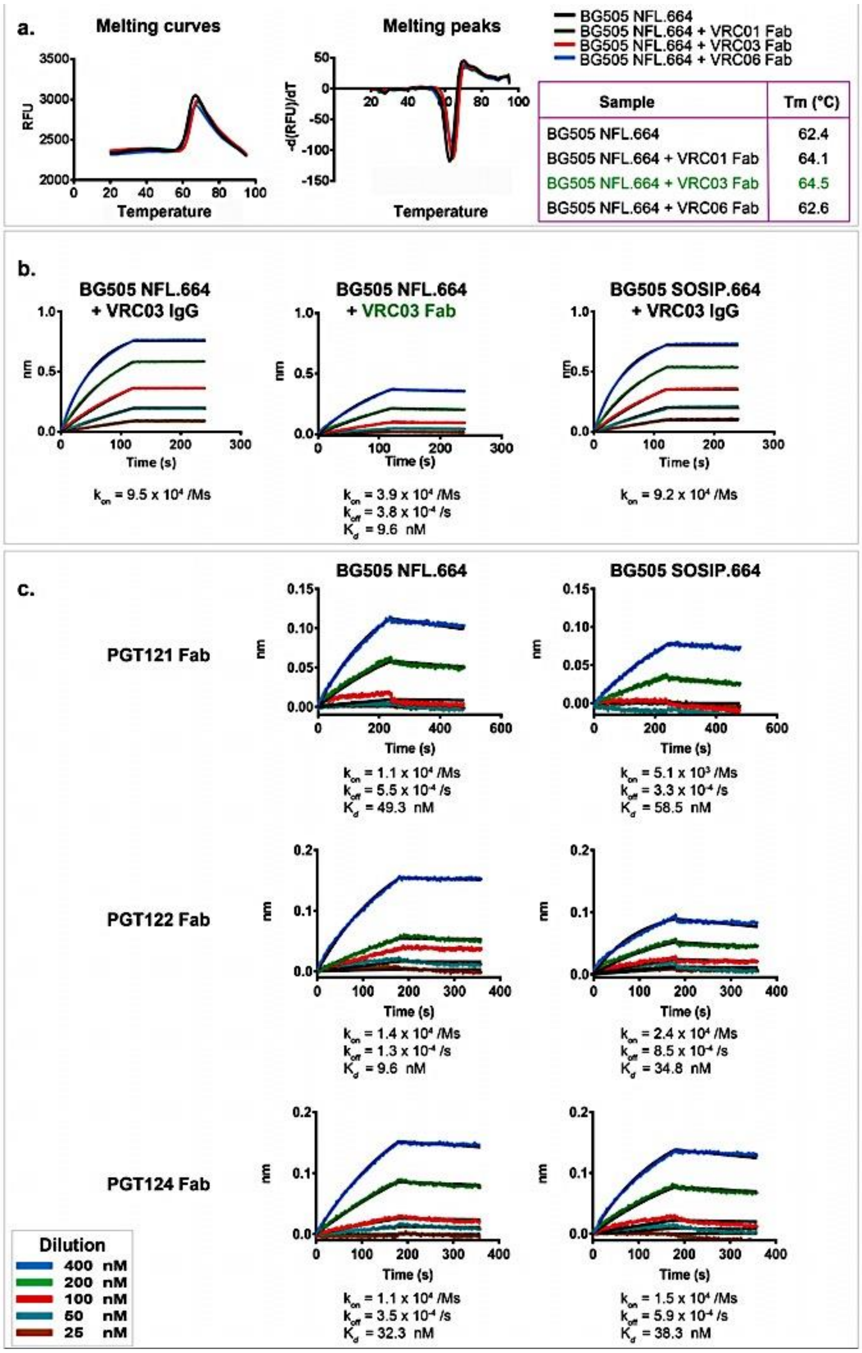

Supplementary Figure 2. Thermostability of BG505 NFL.664 and BG505 SOSIP.664 produced in HEK 293F cells. (a) The raw fluorescence data (left) from thermostability (DSF) studies depicting the melting curves of unbound BG505 NFL.664 and in complex with VRC01, VRC03 and VRC06 Fabs. First derivative of the melting curves of the complexes in (a) on the right show the peak minimum representing the T_m of the complexes. Binding of VRC03 Fab increased the Env trimer thermostability by

2°C and thus was selected for cryo-EM experiments. **(b)** Binding properties of VRC03 IgG and Fab to cleavage-independent and cleaved Env constructs as determined by biolayer interferometry (BLI). Binding characteristics of VRC03 IgG to BG505 NFL.664 is identical to BG505 SOSIP.664 **(c)** Binding properties of PGT121, PGT122 and PGT124 Fabs to cleavage-independent soluble BG505 NFL.664 are comparable to or slightly greater than its SOSIP counterpart. All experiments were performed in duplicate or triplicate.

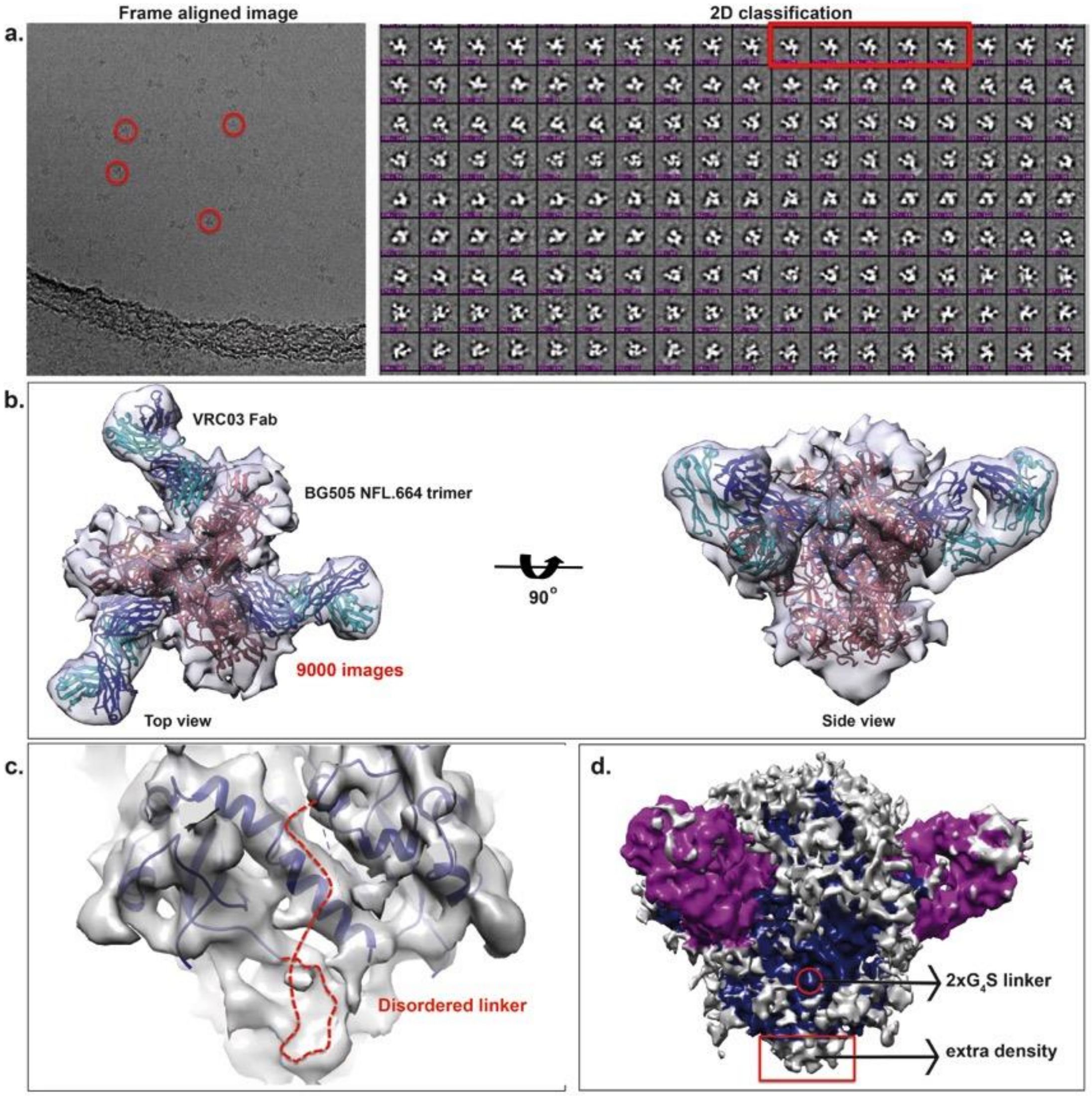

Supplementary Figure 3. High-resolution cryo-EM characterization of BG505 NFL.664 bound to VRC03 Fab. (a) The frame aligned image and the 2D classification of the BG505 NFL.664 in complex with three VRC03 Fabs. **(b)** Top and side views of the cryo-EM density map constructed from 9000 images, fitted with the crystal coordinates of BG505 NFL.664 (this study) with three VRC03 Fabs modeled from PDB 3SE8[1] [https://www.rcsb.org/structure/3se8]). **(c)** The flexible linker (red dotted line) was disordered in the EM map. **(d)** Superposition of the EM maps of BG505 NFL.664 (gray) in complex with VRC03 Fabs (gray) and BG505 SOSIP.664 (blue) in complex with PGV04 Fabs (magenta) (EMD-5779) [http://emsearch.rutgers.edu/atlas/5779_summary.html] indicates the possible location of

the flexible 2xG_4S linker. The extra density seen at the bottom of the NFL map likely corresponds to the 8 residues of the His-tag. Cryo-EM figures were generated using Chimera[1].

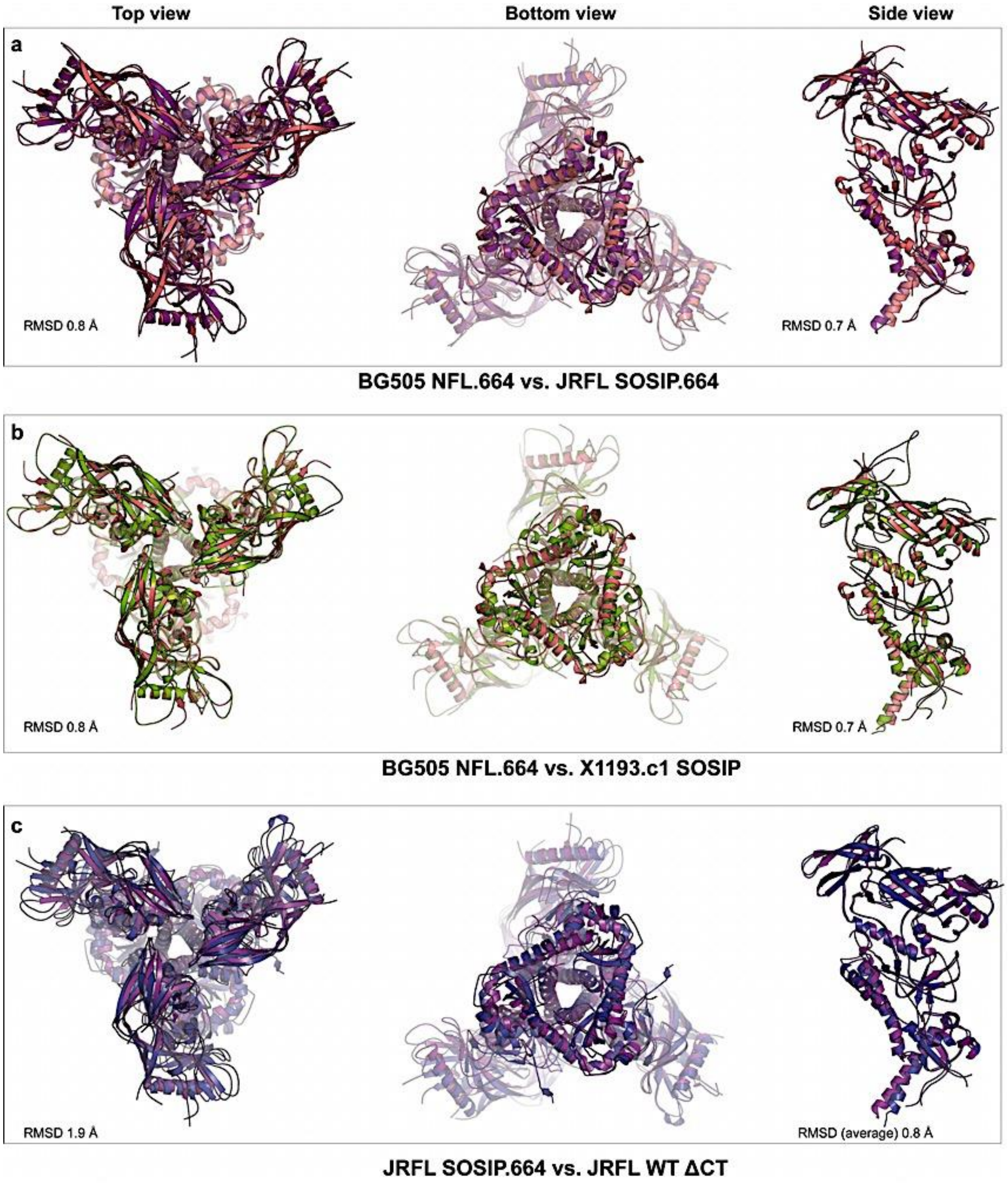

Supplementary Figure 4. Cross-clade structural comparison between two classes of Env designs and different constructs within the same clade. Architecture of the protomer and trimer (bottom and top views) of clade A BG505 NFL.664 (salmon) compared to the cleaved constructs of **(a)** clade B JRFL SOSIP.664 (PDB 5FYK[https://www.rcsb.org/structure/5fyk], purple), and **(b)** clade G isolate X1193.c1 SOSIP.664 (PDB 5FYJ [https://www.rcsb.org/structure/5fyj], green). **(c)** Comparison of two cleaved constructs of varying length from clade B: JRFL SOSIP.664 (purple) and

JRFL WTΔCT (PDB 5FUU [https://www.rcsb.org/structure/5fuu], dark blue). All structures were illustrated using PyMOL[2].

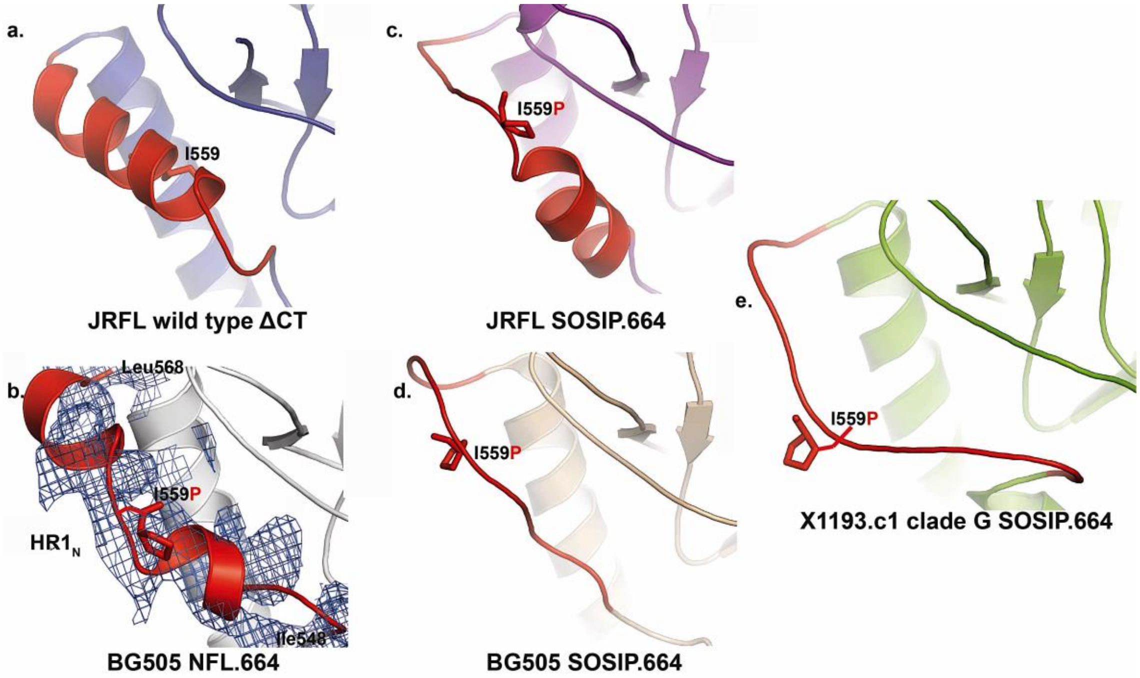

Supplementary Figure 5. Structure of the $HR1_N$ region across clades and designs. The $2F_o$-F_c electron density map (at 1σ level) shows the 20-residue stretch (548-568) at the N-terminus of the HR1 region (red) in various structures. This region in: (**a**) JRFL WTΔCT (PDB 5FUU [https://www.rcsb.org/structure/5fuu]) is completely helical, **(b)** BG505 NFL.664 has a largely helical conformation with I559P mutation disrupting the helix, **(c)** JRFL SOSIP.664 has an N-terminal helix disrupted by the I559P mutation (PDB 5FYK [https://www.rcsb.org/structure/5fyk]), and **(d, e)** tends to be an extended and more disordered loop in other SOSIP structures (clades A and G: PDB 5CEZ [https://www.rcsb.org/structure/5cez] and 5FYJ [https://www.rcsb.org/structure/5fyj], respectively).

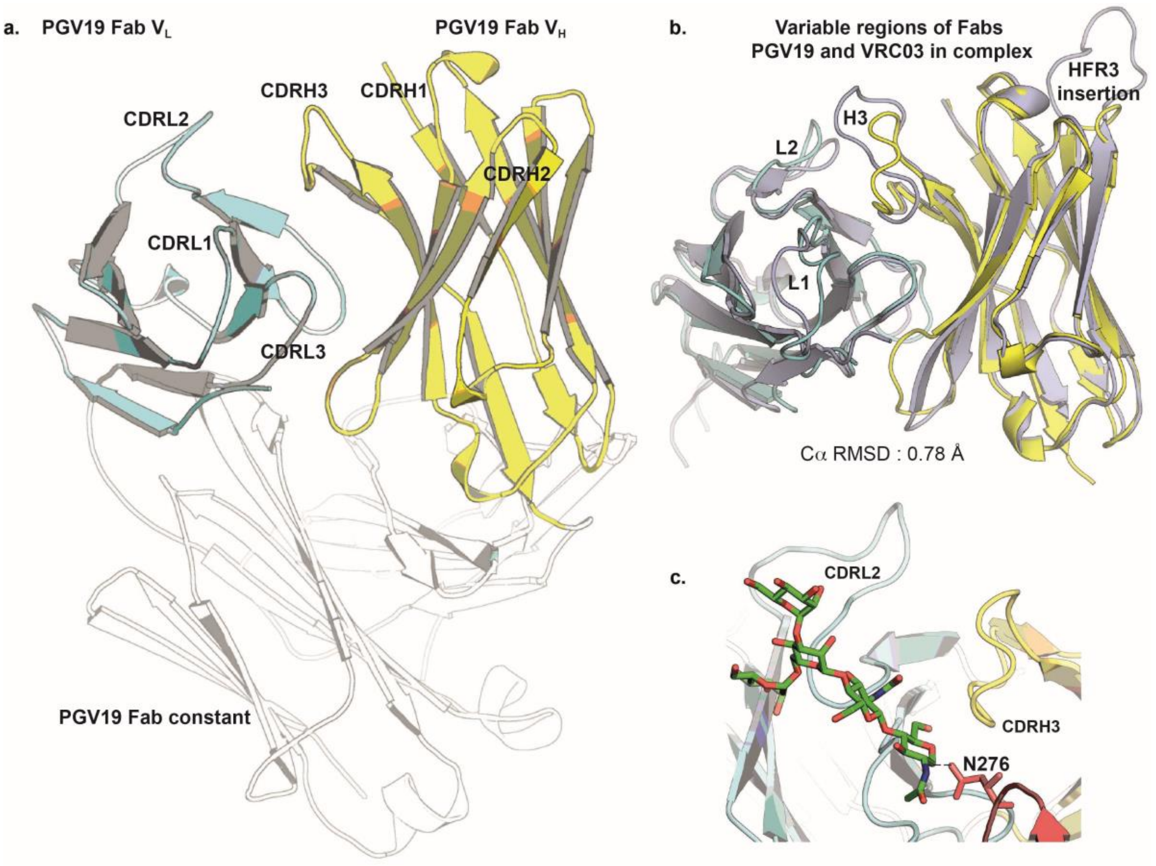

Supplementary Figure 6. Comparison between PGV19 and VRC03 Fabs, and accommodation of N276. (a) The unbound PGV19 Fab structure and its CDR loops with variable light chain in cyan and variable heavy chain in yellow. **(b)** Superimposition of the F*v* regions of PGV19 and VRC03 Fab (slate blue) highlights the similarities in its CDR loops except for CDRL1, CDRL2, CDRH3 and a large insert in HFR3 of VRC03. **(c)** Accommodation of N276 by PGV19.

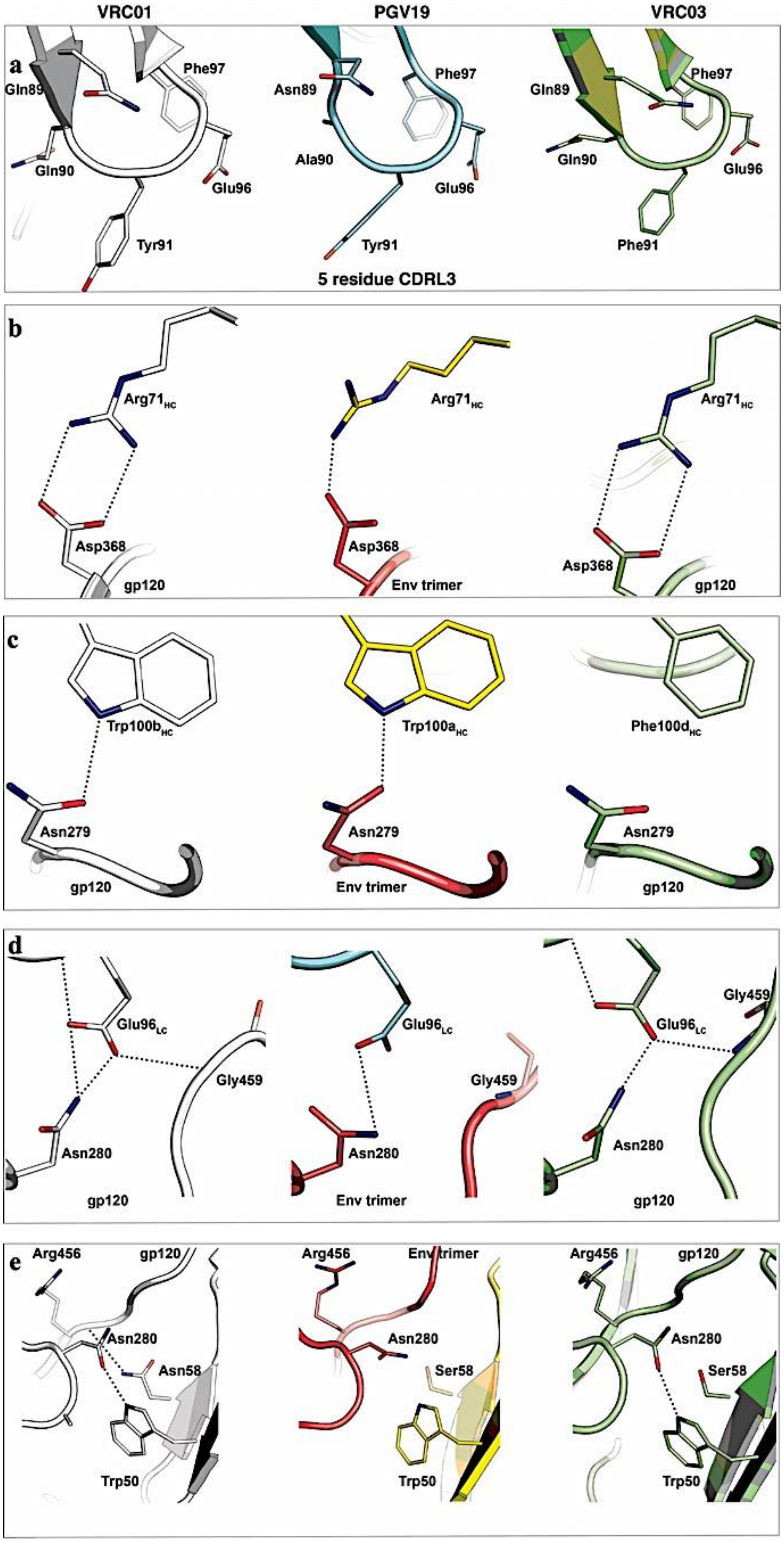
VRC01
PGV19
VRC03
a
Gln89
Phe97
Gln90
Glu96
Tyr91
Asn89
Ala90
Phe97
Glu96
Tyr91
5 residue CDRL3
Gln89
Phe97
Gln90
Glu96
Phe91
b
Arg71HC
Asp368
gp120
Arg71HC
Asp368
Env trimer
Arg71HC
Asp368
gp120
c
Trp100bHC
Asn279
gp120
Trp100aHC
Asn279
Env trimer
Phe100dHC
Asn279
gp120
d
Glu96LC
Gly459
Asn280
gp120
Glu96LC
Gly459
Asn280
Env trimer
Gly459
Glu96LC
Asn280
gp120
e
Arg456
gp120
Asn280
Asn58
Trp50
Arg456
Env trimer
Asn280
Ser58
Trp50
Arg456
gp120
Asn280
Ser58
Trp50

Supplementary Figure 7. Similarity and variation within the VRC01-class signature features and residues in VRC01, PGV19 and VRC03. Representation of **(a)** the conserved 5-residue CDRL3, **(b)** hydrogen bond/salt bridge interaction between $Arg71_{HC}$ and $Asp368_{gp120}$, **(c)** hydrogen bond between $Asn279_{gp120}$ with a Trp (100a/b/d) on CDRH3, **(d)** $Glu96_{LC}$ hydrogen bonds with $Asn280_{gp120}$ and $Gly459_{gp120}$, and **(e)** no hydrogen bonding occurs with the main chain of $Arg456_{gp120}$ due to the presence of a serine at position 58 instead of asparagine (as in VRC01). VRC03 retains a hydrogen bond between Trp50 of CDRH2, which is lost in PGV19. VRC01-gp120 (PDB 3NGB[3] [https://www.rcsb.org/structure/3ngb], 2.68Å resolution) is shown in white, PGV19 (HC: yellow, LC: cyan)-BG505 NFL.664 in salmon, and VRC03-gp120 (PDB 3SE8[4] [https://www.rcsb.org/structure/3se8], 1.89Å resolution) in green. Panels (b) to (e) show potential hydrogen bonds between residues of BG505 NFL.664 and PGV19 (3.39 Å resolution).

Supplementary Table 1. X-ray crystallographic data and refinement statistics

	**PGV19 Fab**	**BG505 NFL.664**
**Data collection**		
Beamline	APS 23-ID B	APS 23-ID D
Detector	Pilatus3 6M	Pilatus3 6M
Wavelength (Å)	1.03317	1.03321
Space group	$P4_32_12$	$P6_3$
Unit cell (*a*, *b*, *c*; Å)	69.78, 69.78, 193.28	161.25, 161.25, 245.65
Resolution range* (Å)	50.00 – 2.50 (2.54 -2.50)	50.00 – 3.39 (3.51 -3.39)
No. of total reflections	136,152 (6,529)	315,116 (30,323)
No. of unique reflections	17,498 (837)	49,063 (4,971)
Redundancy*	7.8 (7.8)	6.4 (6.1)
Completeness* (%)	100.0 (100.0)	98.7 (99.9)
R_{sym} [a*]	16.0 (67.7)	20.6 (>100)
R_{pim} [b*]	6.0 (25.8)	14.5 (55.9)
$\langle I\rangle/\langle \sigma_I\rangle$ *	13.2 (3.1)	6.9 (1.3)
$CC_{1/2}$ [c *]	95.8 (80.2)	71.5 (20.2)
Solvent content (%)	51.4	75.2
**Refinement**		
Reflections used	17,336	48,931
R_{cryst} [d] (%)	20.5	31.1
R_{free} [e] (%)	25.0	32.6
**Model components (asymmetric unit)**		
PGV19 / PGT122	1 / 0	1 / 1
BG505 NFL.664	-	1
Glycerol	4	0
Glycan	-	78
Water	153	-
**B-values (Å^2)**		
Wilson B	31	92
Overall	30	156
NFL/PGV19/PGT122	-	115/ 162 /131
Glycan	-	107
Water	33	-
**Root mean square deviation from ideal values**		
Bond lengths (Å)	0.002	0.005
Bond angles (°)	0.67	0.83
**Ramachandran values**		
Most favored (%)	96.9	96.1
Additional allowed (%)	3.1	3.7
Disallowed (%)	0.0	0.2
**PDB code**	**6AVN**	**6B0N**

* Values in parentheses correspond to the highest resolution shells

[a] $R_{sym} = \Sigma_{hkl} \Sigma_{j=1,N} |\langle I_{hkl}\rangle - I_{hklj}| / \Sigma_{hkl} \Sigma_{j=1,N} |I_{hklj}|$, where the outer sum (hkl) is taken over the unique reflections

[b] $R_{pim} = \Sigma_{hkl}[1/(N-1)]^{1/2} \Sigma_{i=1,N}|I_{hkli} - \langle I_{hkl}\rangle| / \Sigma_{hkl} \Sigma_{i=1,N}|I_{hkli}|$

[e] $CC_{1/2}$ = correlation coefficient of half-datasets[5]

[d] $R_{cryst} = \Sigma_{hkl}||F_{o,hkl}| - k|F_{c,hkl}|| / \Sigma_{hkl}|F_{o,hkl}|$, where $|F_{o,hkl}|$ and $|F_{c,hkl}|$ are the observed and calculated structure factor amplitudes, respectively

[e] R_{free} , as for R_{cryst}, but for a set of reflections (5% of total) omitted from refinement

Supplementary references:

1. Pettersen, E.F. et al. UCSF Chimera—A visualization system for exploratory research and analysis. *J Comput Chem* **25**, 1605-12 (2004).
2. Schrodinger, L. The PyMOL molecular graphics system, version 1.8. (2015).
3. Zhou, T. et al. Structural basis for broad and potent neutralization of HIV-1 by antibody VRC01. *Science* **329**, 811-7 (2010).
4. Wu, X. et al. Focused evolution of HIV-1 neutralizing antibodies revealed by structures and deep sequencing. *Science* **333**, 1593-602 (2011).
5. Karplus, P.A. & Diederichs, K. Linking crystallographic model and data quality. *Science* **336**, 1030-3 (2012)
