# Supplementary material for: Structure of a cleavage-independent HIV Env recapitulates the glycoprotein architecture of the native cleaved trimer
Source: Nat Commun. 2018 May 16;9:1956. doi: 10.1038/s41467-018-04272-y (PMC5955915; doi:10.1038/s41467-018-04272-y)
Supplement: Supplementary file 3 — Description of Additional Supplementary Files [file 41467_2018_4272_MOESM3_ESM.pdf]

Description of Additional Supplementary Files

File Name: Supplementary Movie 1

Description: Tracking the movement of the flexible (2xG_4S) peptide linker during MD simulation of glycosylated BG505 NFL gp140, related to **Figure 3**. In the video, gp120 is colored cyan, gp41 in blue, and the flexible linker in red. VMD[1] was used for visualization of the trajectory.

File Name: Supplementary Data 1

Description: Library of glycan structures identified on BG505 NFL.664 trimer, related to **Figure 5**. Structures were identified and verified by ion mobility mass spectrometry and are represented using the Oxford glycan nomenclature (Oxford) as previously described[2]. (Da: Dalton; Calc: Calculated. Ions are explained in this data file).

File Name: Supplementary Data 2

Description: N-linked glycopeptide compositions of trypsin- and chymotrypsin-digested BG505 NFL.664 trimers produced in HEK 293F cells identified by LC-ESI MS, related to **Figure 5** for site-specific N-glycosylation analysis of BG505 NFL.664**.** Relative quantification of the micro-heterogeneity of 20 N-glycosylation sites of BG505NFL.664 produced in HEK 293F cells. The glycoproteins were enzymatically digested using trypsin or chymotrypsin and analyzed by LC-ESI MS. The bar graph represents the means of two analytical replicates and the pie charts display the overall abundance of oligomannose-type (green) and complex- and hybrid-type (pink) glycans. A glycan library (Supplementary Data 1) generated by ion mobility mass spectrometry was used as the basis for this analysis. Supplementary Data 2 shows the identified glycopeptide peak lists including the intensities of the extracted ion chromatograms used for relative quantification. Identified glycoforms were grouped according to the number of their antennae. The glycan names are delineated as follows and as previously described[3]: Mn = number (n) of mannose residues; An = number (n) of antennae (e.g. A2 = biantennary); Gn = number (n) of galactose residues; F indicates the presence of a core fucose.

References

1. Humphrey, W., Dalke, A. & Schulten, K. VMD - Visual Molecular Dynamics. J. *Molec. Graphics* 14, 33-38 (1996).
2. Harvey, D.J., Merry, A.H., Royle, L., Campbell, M.P. & Rudd, P.M. Symbol nomenclature for representing glycan structures: Extension to cover different carbohydrate types. *Proteomics* **11**, 4291-5 (2011).
3. Behrens, A.J. et al. Composition and antigenic effects of individual glycan sites of a trimeric HIV-1 envelope glycoprotein. *Cell Rep* 14, 2695-706 (2016).
